# Supplementary material for: Studying an antiaromatic polycyclic hydrocarbon adsorbed on different surfaces
Source: Nat Commun. 2018 Mar 22;9:1198. doi: 10.1038/s41467-018-03368-9 (PMC5864723; doi:10.1038/s41467-018-03368-9)
Supplement: Supplementary file 1 — Supplementary Information(PDF 5128 kb) [file 41467_2018_3368_MOESM1_ESM.pdf]

Studying an antiaromatic polycyclic hydrocarbon adsorbed on different surfaces

Majzik et al.

Supplementary Notes

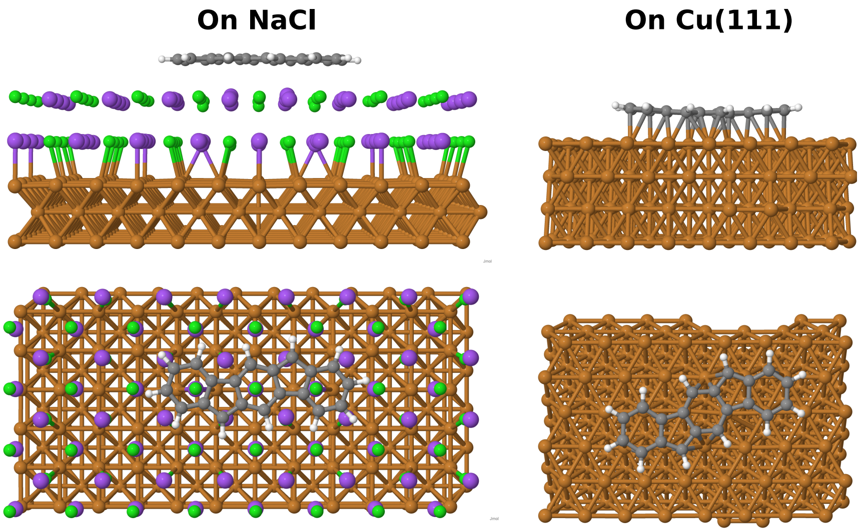

Supplementary Figure 1: Calculated adsorption geometries on NaCl and on Cu(1 1 1).

In agreement with the experimental z^* maps, calculated geometries indicate that **IF** adsorbs planar on NaCl and on Cu(1 1 1) there is a slight increase in the adsorption height at the outer benzene rings compared to the molecular centre. We determined the lateral adsorption-positions of **IF** from variable constant-height AFM images and used them as inputs for DFT calculations.[1] For structural relaxations, on NaCl the Perdew-Burke-Ernzerhof (PBE) exchange-correlation functional was applied[2] with vdW correction.[3] The convergence criteria for the total forces was 10^{-3} eV/Å and for the total energy it was set to 10^{-5} eV. On Cu(1 1 1) the Hybrid functional HSE (Heyd, Scuseria and Ernzerhof) with vdW correction provided the best fit with the experimentally determined adsorption position. A mixing coefficient of 0.8 was used for HSE geometry optimization and the convergence criteria for the total forces was 10^{-3} eV/Å and for the total energy it was set to 10^{-4} eV.

Supplementary Discussion

Generation of indeno[1,2-*b*]fluorene (IF) in solution

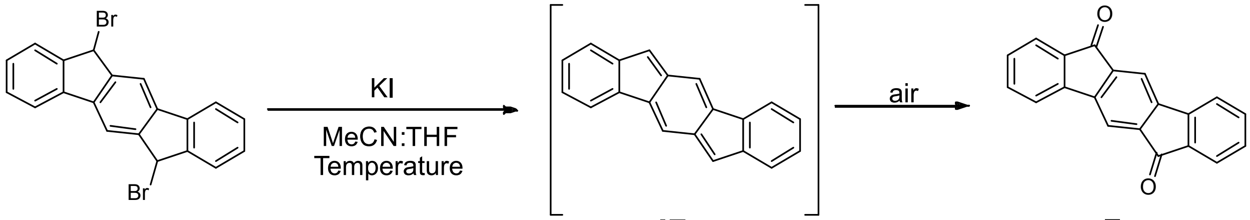

2 **IF** **7**

Supplementary Figure 2: Generation of **IF** in solution.

The reaction of compound **2** in the presence of KI in a MeCN:THF mixture at room temperature led to the formation of dione **7**,[4] presumably via oxidation of intermediate **IF** (Supplementary Figure 2). The reaction was monitored by UV/Vis spectroscopy, showing a transient group of signals with a maximum at 507 nm. At 20 °C, the maximum intensity was reached after 180 s of KI addition (see main manuscript, Figure 2), while after 480 s the signal at 507 nm was not detected anymore. This group of signals was assigned to intermediate **IF** by comparison with the UV/Vis spectrum of stable substituted **IF** derivatives.[5] Among potential intermediates (**IF** and single radicals with different molecular charges) shown in Table 1, the calculated gap of **IF** shows the best agreement with the optically determined gap of 2.33 eV, supporting further that we have **IF** as an intermediate formed during the reaction shown in Supplementary Figure 2.

Supplementary Table 1: Computational and optical data of intermediates

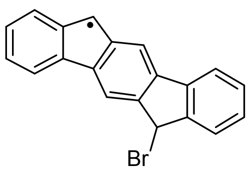

2'

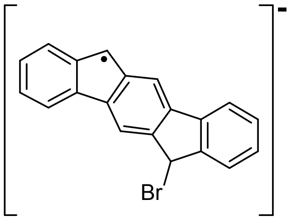

[2']$^-$

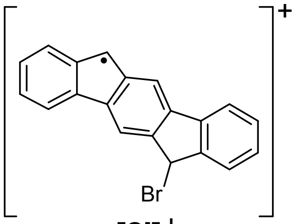

[2']$^+$

	Computational				Optical		
Compound	E_{HOMO}	E_{SUMO}	E_{LUMO}	E_{gap}	λ_{max}	λ_{edge}	E_{gap}
**2**	-5.98		-2.19	3.79	329	345	3.59
**2'**	-5.81	-4.47	-1.74	2.73			
**[2']$^-$**	-1.84		-0.36	1.47			
**[2']$^+$**	-9.43		-8.29	1.14			
**IF**	-5.42		-3.17	2.25	507	534*	2.33*

Hybrid functional HSE (Heyd, Scuseria and Ernzerhof)[6,7] with a mixing coefficient of 0.3 was applied for computational calculations. Wavelengths λ_{max}, λ_{edge} are in nm and energies E_{HOMO}, E_{SUMO}, E_{LUMO}, E_{gap} are in eV. *The optical gap shows the best match with the calculated gap of **IF**.

We performed the reaction shown in Supplementary Figure 2 at different temperatures (5, 10 and 20 °C). Fig. 3 shows the evolution of the absorbance peak at 507 nm after the addition of KI (t = 0). We found that the peak at 507 nm persists longer at lower temperature, suggesting that the lifetime of **IF** increases significantly as the temperature decreases.

Supplementary Figure 4 show the UV/Vis spectra of starting material **2** and oxidation product **7**, respectively.

We also investigated on-surface generation of **IF** from dibromo precursor **2**, which was used for characterisation in solution. On Cu(1 1 1) and on 2ML NaCl **2** debrominated spontaneously during adsorption forming **IF** and Br adatoms (see Supplementary Figure 5).

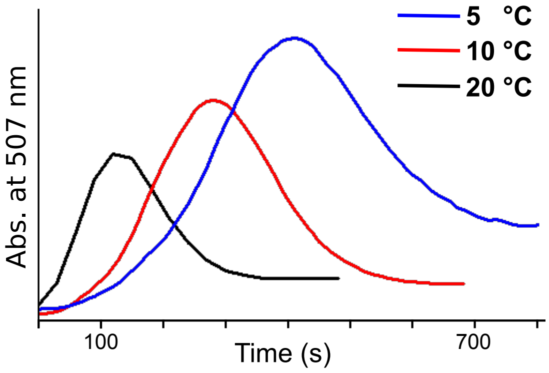

Supplementary Figure 3: Temperature and time dependence of the absorbance peak at 507 nm.

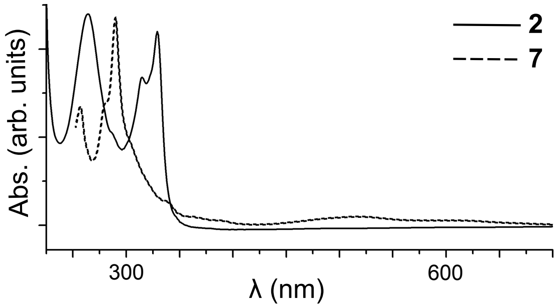

Supplementary Figure 4: UV/Vis spectrum of compounds **2** and **7**.

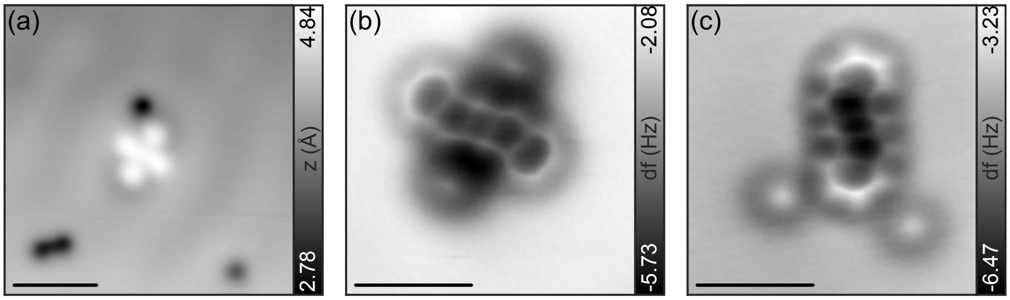

Supplementary Figure 5: (a) Constant current STM image ($I = 2$ pA, $V = 0.1$ V) of **IF** formed by spontaneous debromination of precursor **2** on on 2 ML NaCl. (b, c) Constant-height AFM images of **IF** on 2 ML NaCl (b) and Cu(1 1 1) (c). The STM image was recorded with a metal tip and AFM images were taken with a CO-tip at $\Delta z = 1.5$ Å (b) $\Delta z = -2.3$ Å (c) with respect to the STM set-point of $I = 1.0$ pA and $V = 0.1$ V above the respective substrate. Scale bars, (a) 2 nm, (b, c) 1 nm.

On-surface dehydrogenation of 3

In Supplementary Figure 6 we present a typical example for the dehydrogenation of **3** on Cu(1 1 1) and on 2 ML NaCl. AFM images were taken with a CO-functionalized tip at zero bias ($V = 0$). Height offsets Δz are denoted with respect to an STM set-point of $I = 1$ pA at $V = 0.1$ V above the respective substrate surface. For dehydrogenation a Cu terminated tip was used. The tip was positioned above the center of a precursor molecule **3** at a tip height corresponding to an STM setpoint of $V = 0.1$ V and $I = 1$ pA. On Cu(1 1 1), at opened-feedback loop the tip was retracted by 4.5 Å (z_{off}) and the sample voltage V was increased to 3.5 V for two seconds. We observed a sudden increase in the current after about 1 s in the first and in the second dehydrogenation steps (Supplementary Figure 6b and d, respectively). On 2 ML NaCl the molecule started to change its lateral position already above above 1.5 V.

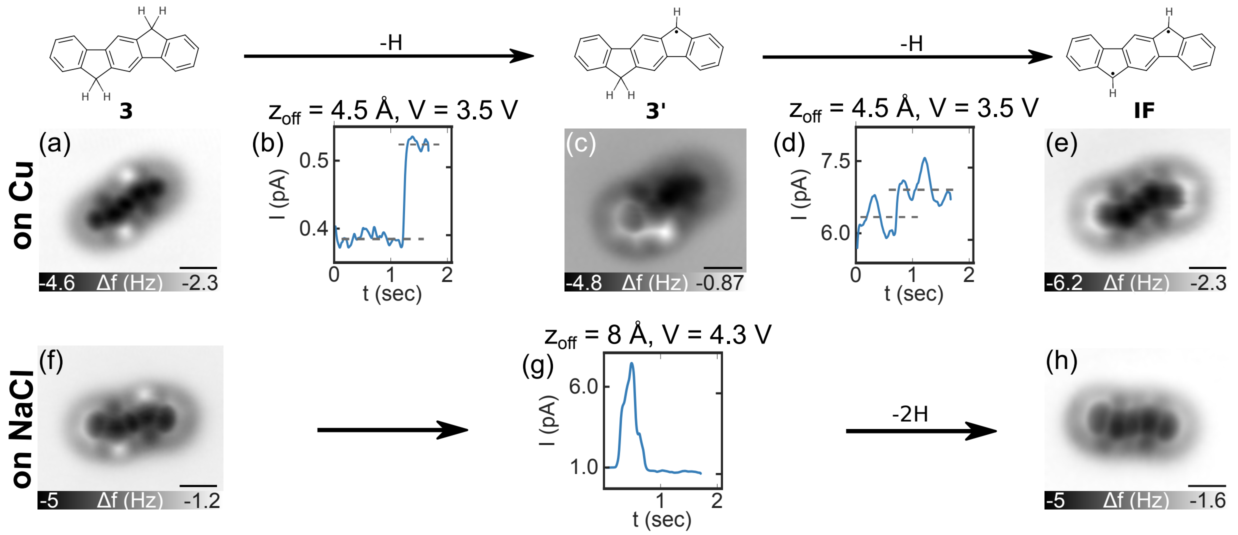

Supplementary Figure 6: On surface generation of indeno[1,2-*b*]fluorene (**IF**) from 6,12-dihydroindeno[1,2-*b*]fluorene (**3**). (a, c, e, f, h) Constant-height AFM images of the precursor molecule **3** (a, f), of radical **3'** (c) and **IF** (e, h) on Cu(1 1 1) (a, c, e) and on 2ML NaCl/Cu(1 1 1) (f, h). Images were taken at (a) $\Delta z = -1.3$ Å, (c) $\Delta z = -2.0$ Å, (e) $\Delta z = -2.5$ Å, (f) $\Delta z = 1.7$ Å and (h) $\Delta z = 1.5$ Å. (b, d, g) $I(t)$ curves recorded during bias treatments. A sudden increase in the current indicates a dehydrogenation event. On 2 ML NaCl the molecule moved laterally out of the tip-sample junction immediately after dissociation of both hydrogens inducing a drop in the current (g). **IF** on NaCl (h) was found typically a few nm displaced from the original adsorption site of the precursor molecule **3** (f). Scale bars, 500 pm.

To dehydrogenate, we typically had to use a larger bias voltage than on Cu(1 1 1) and retract further the tip to limit the tunnelling current (see Supplementary Figure 6g). Below 4 V, **3** normally moved out of the tip-sample junction before a manipulation could have occurred.

Supplementary Methods

All reactions were carried out under argon using oven-dried glassware. TLC was performed on Merck silica gel 60 F254; chromatograms were visualized with UV light (254 and 360 nm). Flash column chromatography was performed on Merck silica gel 60 (ASTM 230-400 mesh). ^{1}H and ^{13}C NMR spectra were recorded at 300 and 75 MHz or 500 and 125 MHz (Varian Mercury 300 or Bruker DPX-500 instruments), respectively. Low-resolution electron impact mass spectra were determined at 70 eV on a HP-5988A instrument. High-resolution mass spectra (HRMS) were obtained on a Micromass Autospec spectrometer. UV/Vis spectra were recorded in a Jasco V-630 spectrophotometer.

Compounds **3** and **6** were prepared following a reported procedure.[8] Commercial reagents and anhydrous solvents were purchased from ABCR GmbH, Aldrich Chemical Co., or Strem Chemicals Inc., and were used without further purification.

Synthesis of 2,2”-dichloro-2’,5’-dimethyl-1,1’:4’,1”-terphenyl (6)

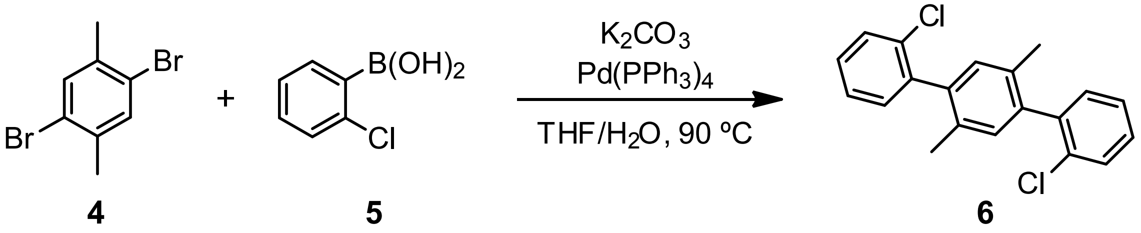

Supplementary Figure 7: Synthesis of terphenyl **6**.[8]

To a deoxygenated solution of 1,4-dibromo-2.5-dimethylbenzene (**4**, 200 mg, 0.76 mmol), (2-chlorophenyl)boronic acid (**5**, 294 mg, 1.90 mmol) and K_2CO_3 (836 mg, 6.00 mmol) in a mixture of THF:H_2O (7:3, 20 mL), $Pd(PPh_3)_4$ (80 mg, 0.069 mmol) was added and the resulting mixture was heated at 90 °C for 16 h. After cooling, the organic layer was separated and the aqueous layer was extracted with Et_2O (3×5 mL). The combined organic extracts were dried over Na_2SO_4, filtered and evaporated under reduced pressure. The crude product was purified by column chromatography (SiO_2, hexane/CH_2Cl_2 5:1) to afford 2,2”-dichloro-

2',5'-dimethyl-1,1':4',1"-terphenyl (**6**) as a colorless solid (239 mg, 97%). ^{1}H NMR (300 MHz, $CDCl_3$) δ: 7.40 (m, 8H), 7.06 (s, 2H), 2.12 (s, 6H) ppm.[8]

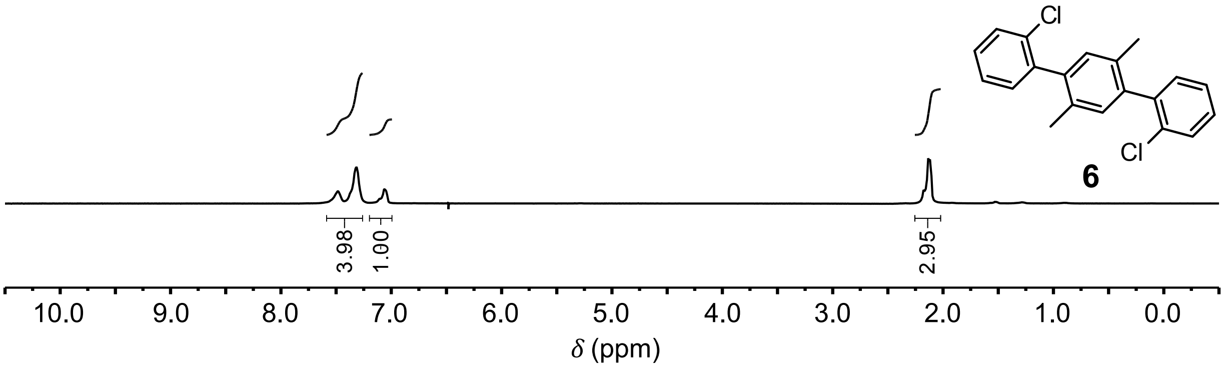

Supplementary Figure 8: ^{1}H NMR of terphenyl **6**

Synthesis of 6,12-dihydroindeno[1,2-*b*]fluorene (3)

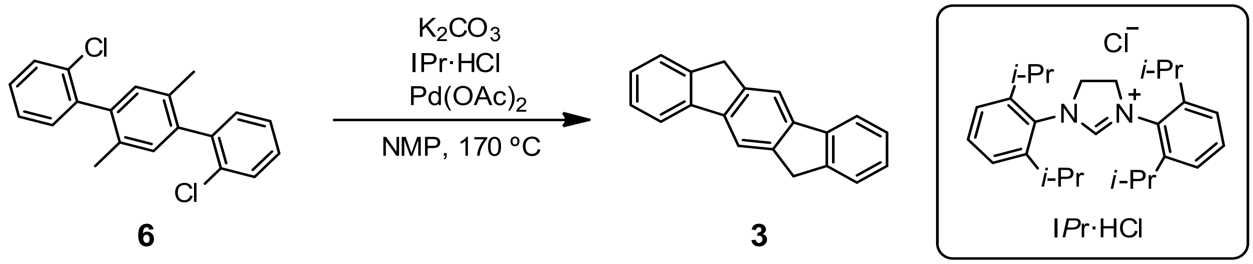

Supplementary Figure 9: Synthesis of dihydroindenofluorene **3**.

A mixture of terphenyl **6** (180 mg, 0.55 mmol), K_2CO_3 (152 mg, 1.10 mmol), IPr · HCl (47 mg, 0.11 mmol), $Pd(OAc)_2$ (12 mg, 0.053 mmol) and NMP (2 mL) was heated at 170 °C for 2 h. After cooling, MeOH (20 mL) was added and the precipitate was collected by filtration. The resulting solid was purified by column chromatography (SiO_2; hexane:CH_2Cl_2 4:1 to 1:1) to afford compound **3** (102 mg, 73%) as a white solid. ^{1}H NMR (300 MHz, $CDCl_3$) δ: 7.95 (s, 2H), 7.81 (d, J = 7.5 Hz, 2H), 7.56 (d, J = 7.4 Hz, 2H), 7.39 (t, J = 7.0 Hz, 2H), 7.30 (td, J = 7.4, 1.2 Hz, 2H), 3.98 (s, 4H) ppm.[8]

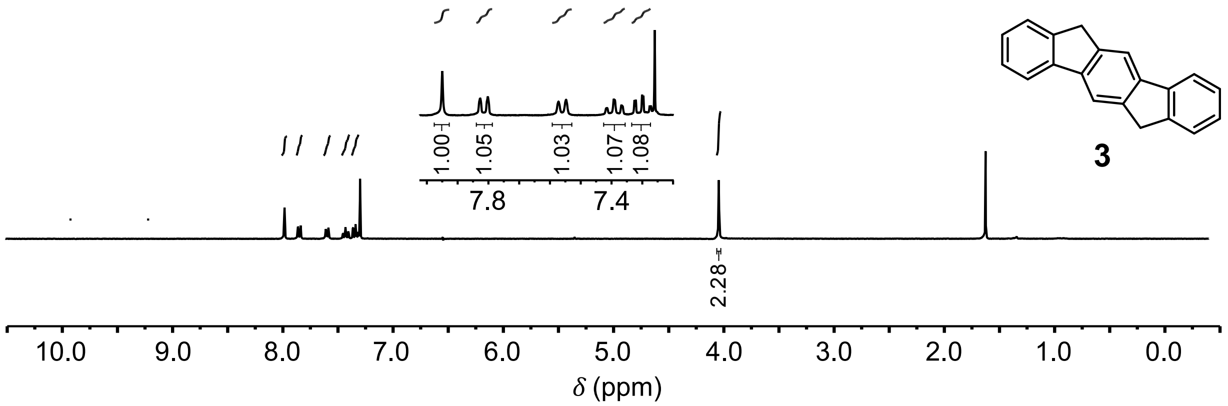

Supplementary Figure 10: ^{1}H NMR of dihydroindenofluorene **3**

Synthesis of 6,12-dibromo-6,12-dihydroindeno[1,2-*b*]fluorene (2)

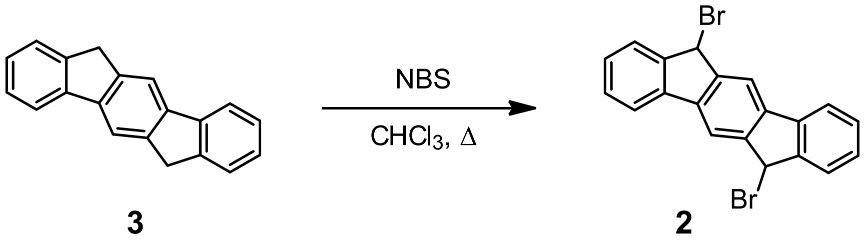

Supplementary Figure 11: Synthesis of 6,12-dibromo-6,12-dihydroindeno[1,2-*b*]fluorene (**2**)

To a solution of **3** (40 mg, 0.16 mmol) and NBS (168 mg, 0.95 mmol) in $CHCl_3$ (5 mL) a catalytic amount of benzoyl peroxide (5 mg approx) was added and the resulting mixture was heated at reflux for 5 h. Then, the solvent was removed under reduced pressure and the residue was washed successively with MeCN (4 × 10 mL) and $CHCl_3$ (2 × 5 mL), affording **2** as a as a yellowish solid (21 mg, 43%,). This solid was identified as a mixture of *syn* and *anti* diastereomers (1:2 ratio). MS (EI) m/z (%): 412 (M^+, 19), 331 (27), 252 (100), 126 (70). HRMS (EI) for $C_{20}H_{12}Br_2$: calculated: 409.9306, found: 409.9286.

Major diastereomer: ^{1}H NMR (353 K, 500 MHz, $C_2D_2Cl_4$) δ: 7.88 (s, 2H), 7.68 (d, J = 7.6 Hz, 2H), 7.62 (d, J = 7.5 Hz, 2H), 7.39 (t, J = 7.4 Hz, 2H), 7.32 (t, J = 7.6 Hz, 2H), 6.03 (s, 2H) ppm. ^{13}C NMR (353 K, 125 MHz, $C_2D_2Cl_4$) δ: 146.11 (2C), 144.71 (2C), 140.40 (2C), 139.52 (2C), 129.69 (2CH), 128.58 (2CH), 126.67 (2CH), 120.59 (2CH), 118.47 (2CH), 46.03 (2CH) ppm.

Minor diastereomer: ^{1}H NMR (353 K, 500 MHz, $C_2D_2Cl_4$) δ: 7.88 (s, 2H), 7.68 (d, J = 7.6 Hz, 2H), 7.62 (d, J = 7.5 Hz, 2H), 7.39 (t, J = 7.4 Hz, 2H), 7.32 (t, J = 7.6 Hz, 2H), 6.02 (s, 2H) ppm. ^{13}C NMR (353 K, 125 MHz, $C_2D_2Cl_4$) δ: 146.05 (2C), 144.74 (2C), 140.43 (2C), 139.57 (2C), 129.69 (2CH), 128.58 (2CH), 126.67 (2CH), 120.59 (2CH), 118.47 (2CH), 46.06 (2CH) ppm.

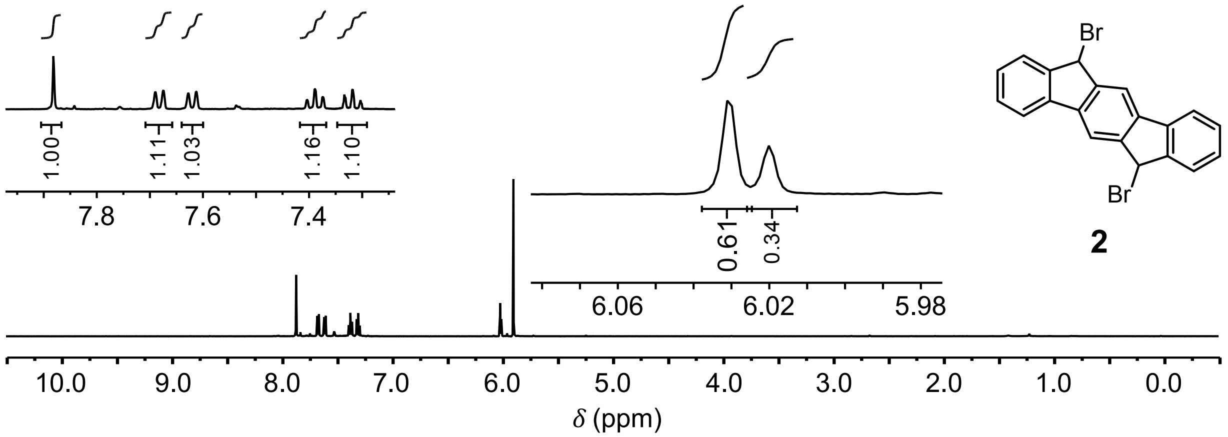

Supplementary Figure 12: ^{1}H NMR of the mixture *syn*- and *anti*-dibromodihydroindenofluorenes **2** (2:1 ratio)

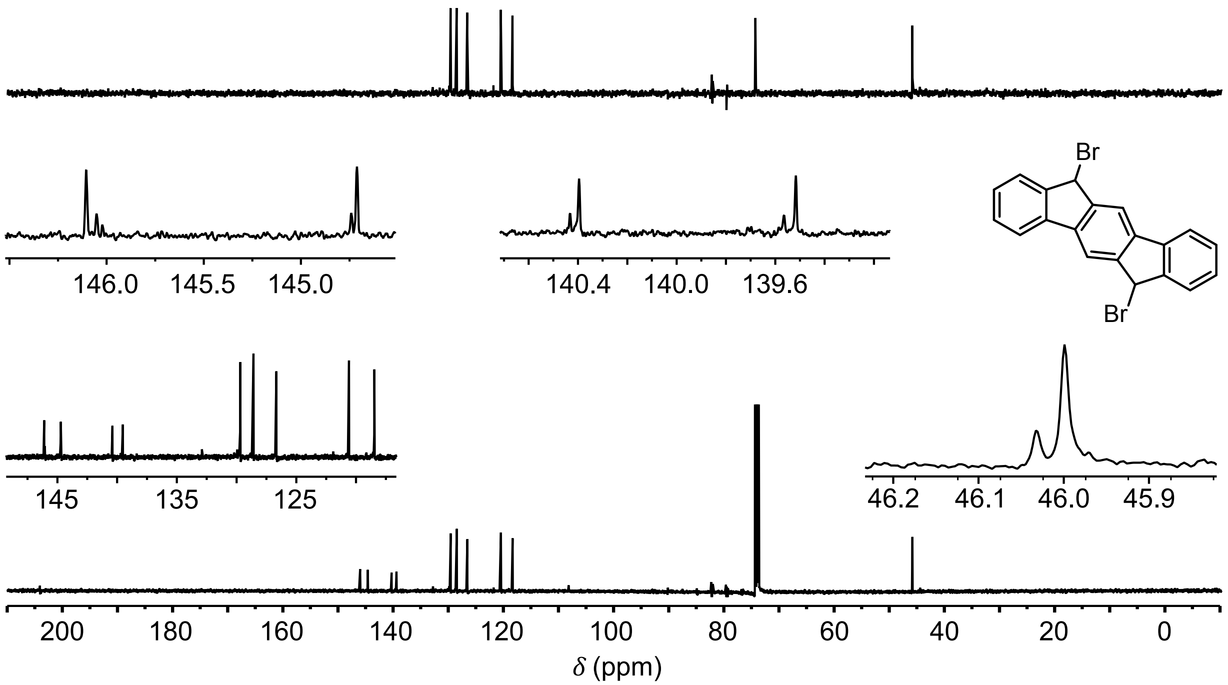

Supplementary Figure 13: ^{13}C NMR of the mixture *syn*- and *anti*-dibromodihydroindenofluorenes **2** (2:1 ratio)

Supplementary References

(1) Schuler, B.; Liu, W.; Tkatchenko, A.; Moll, N.; Meyer, G.; Mistry, A.; Fox, D.; Gross, L. *Phys. Rev. Lett.* **2013**, *111*, 086101.

(2) Perdew, J. P.; Burke, K.; Ernzerhof, M. *Phys. Rev. Lett.* **1996**, *77*, 3865–3868.

(3) Tkatchenko, A.; Scheffler, M. *Phys. Rev. Lett.* **2009**, *102*, 073005.

(4) Liu, T.-P.; Liao, Y.-X.; Xing, C.-H.; Hu, Q.-S. *Org. Lett.* **2011**, *13*, 2452–2455.

(5) Chase, D. T.; Fix, A. G.; Rose, B. D.; Weber, C. D. *Angew. Chem. Int. Ed.* **2011**, *50*, 11103–11106.

(6) Krukau, A. V.; Vydrov, O. A.; Izmaylov, A. F.; Scuseria, G. E. *J. Chem. Phys.* **2006**, *125*, 224106.

(7) Heyd, J.; Scuseria, G. E.; Ernzerhof, M. *J. Chem. Phys.* **2003**, *118*, 8207–8215.

(8) Hsiao, C.-C.; Lin, Y.-K.; Liu, C.-J.; Wu, T.-C.; Wu, Y.-T. *Adv. Synth. Catal.* **2010**, *352*, 3267–3274.
